# Clinical Studies of Diseases of the Eye

**Published:** 1885-07

**Authors:** 


CLINICAL STUDIES ON DISEASES OF THE EYE, INCLUDING THOSE OF THE CONJUNCTIVA, CORNEA, SCLEROTIC, IRIS, AND CILIARY BODY. *By* DR. FERDINAND RITTER VON ARLT, *Professor of Ophthalmology in Vienna. Translated by* LYMAN WARE, M. D., *Surgeon to the Illinois Charitable Eye and Ear Infirmary; Ophthalmic Surgeon to the Presbyterian Hospital and to the Protestant Orphan Asylum, Chicago. Philadelphia:* P. BLAKISTON, SON & CO., *1012 Walnut street. Chicago:* W. T. KEENER, *96 Washington street.*

This is a most excellent translation of a very admirable work. Professor Arlt holds an exceptionally high position in the estimation of all students of ophthalmology throughout the world as a clinical lecturer.

He is the author of a very noteworthy treatise on diseases of the eye, published so many years ago (thirty-five), almost at the dawn of the wonderful development of modern ophthalmic science, that it is now almost obsolete, but still a very interesting and instructive book.

The present work, limited to the tissues indicated on the title page, is an expression of Professor Arlt's remarkably long and extensive experience.

One could wish the author and translator would place in the hands of the American student a complete series of clinical lectures on the remaining tissues of the eye.

The mechanical work of the volume is in harmony with the high character of its scientific and literary merits. E. L. H.

